# Supplementary material for: Heparin-based hydrogel scaffolding alters the transcriptomic profile and increases the chemoresistance of MDA-MB-231 triple-negative breast cancer cells
Source: Biomater Sci. 2020 Feb 13;8(10):2786–96. doi: 10.1039/c9bm01481k (PMC7497406; doi:10.1039/c9bm01481k)
Supplement: Supplementary file 2 [file BM-008-C9BM01481K-s002.zip › Supplementary File 4/HP-BhydrogelvControl/Pathways/my_analysis.Gsea.1545201079907/index.html]

Index for xtools.gsea.Gsea my\_analysis.Gsea.1545201079907

### GSEA Report for Dataset expr

#### Enrichment in phenotype: **HYD (2 samples)**

- 15 / 50 gene sets are upregulated in phenotype **HYD**- 4 gene sets are significant at FDR < 25%- 1 gene sets are significantly enriched at nominal pvalue < 1%- 4 gene sets are significantly enriched at nominal pvalue < 5%- Snapshot of enrichment results- Detailed enrichment results in html format- Detailed enrichment results in excel format (tab delimited text)- Guide to interpret results

#### Enrichment in phenotype: **CONTROL (2 samples)**

- 35 / 50 gene sets are upregulated in phenotype **CONTROL**- 30 gene sets are significantly enriched at FDR < 25%- 19 gene sets are significantly enriched at nominal pvalue < 1%- 26 gene sets are significantly enriched at nominal pvalue < 5%- Snapshot of enrichment results- Detailed enrichment results in html format- Detailed enrichment results in excel format (tab delimited text)- Guide to interpret results

#### Dataset details

- The dataset has 19199 features (genes)- No probe set => gene symbol collapsing was requested, so all 19199 features were used

#### Gene set details

- Gene set size filters (min=15, max=500) resulted in filtering out 0 / 50 gene sets- The remaining 50 gene sets were used in the analysis- List of gene sets used and their sizes (restricted to features in the specified dataset)

#### Gene markers for the **HYD** *versus* **CONTROL** comparison

- The dataset has 19199 features (genes)- # of markers for phenotype **HYD**: 10461 (54.5% ) with correlation area 57.8%- # of markers for phenotype **CONTROL**: 8738 (45.5% ) with correlation area 42.2%- Detailed rank ordered gene list for all features in the dataset- Heat map and gene list correlation  profile for all features in the dataset

#### Global statistics and plots

- Plot of p-values *vs.* NES- Global ES histogram

#### Other

- Parameters used for this analysis

#### Comments

- There were duplicate row identifiers in the specified dataset. One id was arbitarilly choosen. Details are below
  Generally, this is OK, but if you want to avoid this automagic, edit your dataset so that all row ids are unique
  # of row ids in original dataset: 19263
  # of row UNIQUE ids in original dataset: 19199
  # The duplicates were
  Y\_RNA
  Y\_RNA
  Y\_RNA
  Y\_RNA
  Y\_RNA
  Y\_RNA
  Y\_RNA
  Y\_RNA
  Y\_RNA
  Y\_RNA
  Y\_RNA
  Y\_RNA
  Y\_RNA
  Y\_RNA
  Y\_RNA
  Y\_RNA
  Y\_RNA
  Y\_RNA
  Y\_RNA
  Y\_RNA
  Y\_RNA
  Y\_RNA
  Y\_RNA
  Y\_RNA
  Y\_RNA
  Y\_RNA
  Y\_RNA
  U3
  ELFN2
  TMSB15B
  U1
  BMS1P4
  COG8
  U1
  pRNA
  U2
  U1
  7SK
  U2
  U2
  H2BFS
  U2
  pRNA
  U2
  U1
  U2
  pRNA
  Y\_RNA
  Y\_RNA
  pRNA
  U2
  Metazoa\_SRP
  Metazoa\_SRP
  Metazoa\_SRP
  Y\_RNA
  pRNA
  U2
  U1
  U2
  U1
  U2
  U2
  pRNA
  MATR3- Timestamp used as random seed: 1545201081533

---

Report: my\_analysis.Gsea.1545201079907.rpt   by user: hdang

xtools.gsea.Gsea [Wed, Dec 19, '18 0 AM 31]

Website: www.gsea-msigdb.org/gsea
Questions & Suggestions: Contact page
